# The state of loneliness and social isolation research: current knowledge and future directions

**DOI:** 10.1186/s12889-023-15967-3

**Published:** 2023-06-01

**Authors:** Harry Owen Taylor, Thomas K.M. Cudjoe, Feifei Bu, Michelle H. Lim

**Affiliations:** 1 Factor-Inwentash Faculty of Social Work, University of Toronto, Toronto, Canada; 2 Division of Geriatric Medicine and Gerontology, Johns Hopkins University School of Medicine, Baltimore, USA; 3 Research Department of Behavioural Science and Health, Insititute of Epidemiology & Health Care, University College London, London, England; 4 Prevention Research Collaboration, Sydney School of Public Health, Charles Perkins Centre, The University of Sydney, Sydney, NSW Australia; 5 Iverson Health Innovation Research Institute, Swinburne University of Technology, Victoria, Australia

## Abstract

In this editorial, we consider the current state of loneliness and social isolation research around the world, including knowledge gaps in the empirical literature.

## Main text

For centuries, scholars have examined how social conditions influence human relationships and how these relationships influence health—from cell to society [1, 2]. Two important features of research on social relationships include loneliness and social isolation. Loneliness is defined as a perceived/subjective condition in which an individual is dissatisfied with the quality and/or quantity of their social relationships [3]. Social isolation is an objective condition characterized by a lack of contact with other people and being disengaged from groups and social activities [3]. Loneliness and social isolation are sometimes misconstrued as the same phenomena in public discourse and media; however, previous research has shown they are distinct psychosocial constructs that are weakly to moderately correlated with each other [3]. As a result, it is possible to be lonely and socially isolated, lonely but not isolated, and isolated but not lonely. Additionally, loneliness and social isolation are mechanistically associated with different health outcomes [4].

Findings from empirical studies indicate increases in loneliness and/or social isolation are independently associated with poorer health [5]. Loneliness and social isolation have a mortality risk similar to cigarette smoking, alcohol consumption, physical inactivity, and obesity. Other health outcomes associated with these pernicious conditions include cardiovascular disease, dementia and cognitive decline, and worsening anxiety and depressive symptoms to name a few [5]. Perhaps most notable about this research is the consistency of findings, especially given the multitude of methods in operationalizing loneliness and social isolation, and the diversity of populations and contexts/settings in which these issues are studied [5].

Rates of loneliness and social isolation vary around the world. A recent meta-analysis on country-level differences found loneliness in adolescents was lowest in Southeast Asian countries and highest in Eastern Mediterranean countries [6]. From within Europe, loneliness was highest in Eastern European countries and lowest in Northern European countries [6]. Lim and colleagues [7] found 34% of adults in Australia were lonely, with 21% having episodic loneliness and 13% having chronic loneliness. In the United States, 43% of adults felt they lack companionship, 43% felt that their relationships are not meaningful, 43% felt isolated from others, and 39% no longer feel close to anyone [8]. There are fewer studies of country-level differences in social isolation for the general population; however, in Australia, 17% of the general population were classified as socially isolated, with 13% having episodic and 4% having chronic isolation [7]. Moreover, studies focused on the prevalence of social isolation are often conducted among older adults. A recent Canadian survey, for example, found approximately one out of every four older adults were socially isolated [9]. The COVID-19 pandemic has also brought issues of loneliness and isolation to the fore, especially given the enforcement of social distancing policies from local and federal governments. Evidence on the prevalence rates of loneliness and isolation during the COVID-19 pandemic are mixed with some finding increased rates, and others finding no difference to before the pandemic [10–12].

Given the negative health outcomes and overall prevalence of loneliness and social isolation around the world, we contend these are global public health issues. In many countries, there is investment in policy to ameliorate loneliness and social isolation. Notable movements to address loneliness and isolation include the Campaign to End Loneliness in the United Kingdom, Ending Loneliness Together in Australia, the Foundation for Social Connection in the United States, the World Health Organization’s Social Isolation and Loneliness initiatives, and the Global Initiative on Loneliness and Connection [13]. Other countries [5] have commissioned a body of expert scientific knowledge and policy work to better understand these issues. But loneliness and social isolation are not issues constrained to developed countries. In prevalence studies, these issues occur all over the world [6, 14]; hence global policy and advocacy is sorely needed.

Nevertheless, there are notable gaps in the loneliness and social isolation research literature. There is substantially less research on loneliness and isolation among certain racial/ethnic groups, immigrant communities, diverse gender identities and sexual orientations, disability and neurodivergent population, populations with severe mental illness, people living in poverty, and other social/cultural groups. This is important given those at the greatest social disadvantage and marginalization may have the highest rates of loneliness and social isolation, and may also have heightened risk for becoming lonely and/or isolated [15].

Measurement/operationalization of loneliness and social isolation is also a topic of debate. If loneliness and social isolation are multidomained, how many types of loneliness and isolation are there? What items are important to include to accurately prove the psychometric reliability and validity of loneliness and isolation measurement tools, and how do we ensure that these tools are invariant across age? How do we know if loneliness and social isolation are perceived similarly across cultures? What novel methods exist for measuring loneliness and isolation? How often should we collect data on loneliness and social isolation in longitudinal studies to adequately capture fluctuations and temporal changes? Additionally, there is limited conceptual and empirical work on understanding the interrelationship(s) between loneliness and isolation [3]. This work would be useful for determining the causal mechanisms in which some individuals become lonely and/or isolated, for further understanding how loneliness and isolation influence health and wellbeing, and for the development of evidence-based interventions to address these psychosocial issues.

Lastly, it is important to use this knowledge to inform policy and interventions. What type of interventions, from individual-focused to societal-level, are most impactful, sustainable and/or cost-efficient? Should we use different types of interventions for preventing the onset of loneliness and isolation (primary prevention) versus mitigating these conditions among those who are chronically lonely and isolated (tertiary prevention)? And how do we scale-up these interventions to inform applied clinical or community practice and change public opinion/perceptions on loneliness and social isolation?

The aim of the BMC Public Health collection on loneliness and social isolation is to further our understanding of these psychosocial issues. We hope to propagate this collection of articles to advance research, practice, advocacy, and policy efforts by researchers, scientists, clinicians, policy-makers, community-based and non-profit organizations, governments, and the lay public around the world to facilitate greater social connection for better health and wellbeing for all.

## Data Availability

N/A.

## Declarations

### Authors’ information (optional)

N/A.
